# 

**Published:** 1838-10-01

**Authors:** 


### CONTENTS

OF THE

### MEDICO-CHIRURGICAL REVIEW,

### No. LVIII. OCTOBER 1, 1838,

### [BEING No. XVIII. OF A DECENNIAL SERIES.]

### REVIEWS.

### I.

### TRANSACTIONS OF SOCIETIES.

1. Medico-Chirurgical Transactions. Volume the Twenty-first .. .. ..
2. The Transactions of the Provincial Medical and Surgical Associations. Part II. Volume VI. .. .. .. .. .. .. .. .. .. .. .. .. .. .. .. 353

   1. Remarks on Malignant Diseases of the Skin of the Face. By CÆSAR HAWKINS .. .. .. .. .. .. .. .. .. .. .. .. .. .. . 355
   2. On the peculiar Symptoms occurring in some Cases of Enlarged Liver. By JOHN G. MALCOLMSON, Esq. .. .. .. .. .. .. .. .. .. .. .. 360
   3. On a successful Plan of arresting the Destruction of the Transparent Cornea from Acute Purulent Inflammation of the Conjunctiva. By F. TYRRELL, 363
   4. A Case of Hydatid of the Liver successfully tapped. By WILLIAM TRAVERS COX, M.D. .. .. .. .. .. .. .. .. .. .. .. .. .. .. 368
   5. On the Use of Arsenic in some Affections of the Uterus. By HENRY HUNT, Esq. .. .. .. .. .. .. .. . .. .. .. .. .. 370
   6. Removal of the Clavicle, with a Tumor situated in that Bone. By BENJAMIN TRAVERS, F.R.S. .. .. .. .. .. .. .. .. .. .. .. .. .. .. 373
   7. Case of Excision of the entire Lower Jaw ; with Observations. By J. G. PERRY 376
   8. Nervous Affections peculiar to Young Women, &c. By JOHN WILSON, M.D. 377
   9. A description of an Instrument for closing Vesico-vaginal and Recto-vaginal Fistulæ, invented by Mr. William Beaumont .. .. .. .. .. .. 383
   10. Observations on the Constitution of the Urine. By JOHN BOSTOCK, M.D. F.R.S. .. .. .. .. .. .. .. .. .. .. .. .. .. .. .. .. 383
   11. Report of 20 Cases of Malignant Cholera on board the Dreadnaught. By GEORGE BUDD, M.D., and GEORGE BUSH, Esq. .. .. .. .. .. .. .. 386
   12. On the Treatment of Hypertrophy of the Heart, &c. By THOMAS SALTER, Esq. F.L.S. .. .. .. .. .. .. .. . .. .. .. .. .. .. 395
   13. Two Cases of Gangrene of the Lungs. By WILLIAM ENGLAND, M.D. .. 397
   14. A Case of partial Ectopia Cordis, and Umbilical Hernia. By JOHN O'BRYEN, M. D. .. .. .. .. .. .. .. .. .. .. .. .. .. .. .. .. .. 399
   15. Extirpation of the Eye, on account of a Tumor developed within the Optic Sheath. By R. MIDDLEMORE, Esq. .. .. .. .. .. .. .. .. .. 403

### II.

Transactions of the Medical and Physical Society of Calcutta. Vol. VIII. Part I. .. 405

1. Case of Ranula, in which the Left Submaxillary Gland was extirpated ; with Remarks. By T. G. MALCOLMSON, Esq. .. .. .. .. .. .. .. .. 405

2. A Case of ulcerated Stricture of the Œsophagus, communicating with the Trachea. By A. R. LINDESAY, Esq. .. .. .. .. .. .. .. .. .. 407

3. Case of extensive Liver Abscess, successfully opened. By ARCHIBALD COLQUHOUN .. .. .. .. .. .. .. .. .. .. .. .. .. .. .. .. 409

4. Dislocation of the Os Humeri, reduced after a month and four days .. .. 409

5. Operation performed in Persia for the Removal of Opacity of the Cornea. By S. M. GRIFFITHS, Esq. .. .. .. .. .. .. . .. .. .. .. 409

### III.

Des Maladies Mentales, considerées sur les Rapports Medical, Hygienique, et Medico-Legal. Par E. ESQUIROL.

On Mental Disorders, considered in a Medical, Hygienic, and Medico-legal point of view. By E. ESQUIROL .. .. .. .. .. .. .. .. .. .. .. .. .. .. .. 410
1. Symptoms of Insanity .. .. .. .. .. .. .. .. .. .. .. .. .. .. 411
2. Causes of Insanity .. .. .. .. .. .. .. .. .. .. .. .. .. .. .. 413

IV.

DISEASES OF FEMALES.

Outline of the principal Diseases of Females. By FLEETWOOD CHURCHILL, M.D. .. 417
1. Phlegmonous Inflammation of the External Labia Pudendi .. .. .. .. .. 420
2. Inflammation of the Mucous Membrane of the Vulva .. .. .. .. .. .. 422
3. Inflammation of the Mucous Membrane of the Vagina, or Vaginal Leucorrhœa 424
4. Inflammation of the Glandular Structure of the Mucous Membrane covering the cervix uteri.. .. .. .. .. .. .. .. .. .. .. .. .. .. .. .. 426
5. Granular Inflammation of the Mucous Membrane of the Cervix Uteri.. .. 427
6. Inflammation of the Mucous Membrane of the Uterus; or Uterine Leucorrhœa .. .. .. .. .. .. .. .. .. .. .. .. .. .. .. .. .. .. 427
7. Inflammation of the Substance of the unimpregnated Uterus .. .. .. 430
8. Inflammation of the Fallopian Tubes .. .. .. .. .. .. .. .. .. .. 431
9. Inflammation of the Ovaries .. .. .. .. .. .. .. .. .. .. .. .. .. 432
10. Simple Ulceration of the Cervix Uteri .. .. .. .. .. .. .. .. .. .. 433
11. The corroding Ulcer of the Uterus .. .. .. .. .. .. .. .. .. .. .. 435

V.

De l'Influence des Climats sur l'Homme. Par P. FOISSAC.
On the Influence of Climate on Man. By P. FOISSAC, M.D... .. .. .. .. .. .. 437

VI.

The Cyclopædia of Practical Surgery. Edited by W. B. COSTELLO, M.D. Part III. .. 448
1. Aneurysm .. .. .. .. .. .. .. .. .. .. .. .. .. .. .. .. .. .. 448
2. Angeioleucitis .. .. .. .. .. .. .. .. .. .. .. .. .. .. .. .. .. 452

VII.

Essai sur la Neuralgie du Grand Sympathetique. Par A. SEGOND, D.M.P. .. .. .. 462

VIII.

Counter-irritation, its Principles and Practice, illustrated by One Hundred Cases, &c. By A. B. GRANVILLE, M.D. .. .. .. .. .. .. .. .. .. .. .. .. .. .. 471

IX.

Grundriss der Pathologischen Semeiotik, zum Gebrauche bei Vorlesungen. Von A. F. SCHILL, M.D.

Outlines of Pathological Semeiology .. .. .. .. .. .. .. .. .. .. .. .. .. 477

1. Signs from the Parts of Generation .. .. .. .. .. .. .. .. .. .. .. 485

X.

The Life of Edward Jenner, M.D. &c. &c. By JOHN BARON, M.D. .. .. .. .. 488

### PERISCOPE.

### Bibliographical Notices.

1. The Influence of Religion on Health, and the Physical Welfare of Mankind. By Amariah Brigham, M.D. .. .. .. .. .. .. .. .. .. .. .. .. .. .. .. 529
2. Buxton and its Waters—their Mechanical Properties and General Effects. By W. H. Robertson, M.D. .. .. .. .. .. .. .. .. .. .. .. .. .. .. .. 531
3. Practical Observations on Hysteria, especially relating to its Organic Character. By John Pritchard, M.R.C.S. .. .. .. .. .. .. .. .. .. .. .. .. 534
4. A Fact in the Natural History of Children, hitherto unobserved. By John Gardner, Surgeon .. .. .. .. .. .. .. .. .. .. .. .. .. .. .. .. .. .. 537
5. Practical Compendium of the Materia Medica, with numerous Formulæ. By Alexander Ure, M.D. .. .. .. .. .. .. .. .. .. .. .. .. .. .. .. .. 539
6. The First Principles of Medicine. By Archibald Billing, M.D. .. .. .. .. 541
7. Observations on the Management of Mad-Houses. By Caleb Crowther, M.D. .. 542
8. A Treatise on the Nature and Treatment of Hooping-Cough, &c. By George Hamilton Roe, M.D... .. .. .. .. .. .. .. .. .. .. .. .. .. .. .. .. 545
9. Anatomical Tables. By Mr. T. Nunneley .. .. .. .. .. .. .. .. .. .. .. 547
10. Chemistry of Organic Bodies. By Thomas Thomson, M.D. .. .. .. .. .. 548

### Spirit of the British and American Periodicals.

1. Dr. Gray's Utero-Abdominal Supporter .. .. .. .. .. .. .. .. .. .. .. .. 550

2. Dr. Johnson's Case of Diabetes Mellitus .. .. .. .. .. .. .. .. .. .. .. 552

3. Climate of Torquay .. .. .. .. .. .. .. .. .. .. .. .. .. .. .. 554

4. Nitrate of Silver in Chronic Diseases.. .. .. .. .. .. .. .. .. .. .. 554

5. On the State of the Pupil in Typhus .. .. .. .. .. .. .. .. .. .. 555

6. Beneficial Effects of Belladonna in Puerperal Mania.. .. .. .. .. .. .. 556

7. Recovery of Natives of India from Accidents .. .. .. .. .. .. .. .. 557

8. Dr. Davy on the Fluid in the Vesiculæ Seminales of Man .. .. .. .. .. 558

9. Liabilities of the Population of England and France to Hernia .. .. .. .. 560

### Spirit of the Foreign Periodicals, &c.

1. Some Observations on the Changes produced in the Blood by Medicinal Substances. By C. G. Mitscherlich .. .. .. .. .. .. .. .. .. .. .. .. 561

2. M. Raspail on those Diseases which may be the Work of Insects, and on their Treatment .. .. .. .. .. .. .. .. .. .. .. .. .. .. .. .. 562

3. Scientific Notices in Natural History .. .. .. .. .. .. .. .. .. .. 565

4. On the Influence of the Cerebellum and Spinal Marrow on Erections of the Penis. 567

5. Researches on the Cause of the Abnormal Auscultatory Sounds in the Large Arteries, &c. By M. Beau.. .. .. .. .. .. .. .. .. .. .. .. 569

6. On Sudden Death, occasioned by a Spontaneous Development of Fluid in the Blood, &c... .. .. .. .. .. .. .. .. .. .. .. .. .. .. .. 572

7. M. Velpeau on the Accidental Introduction of Air into the Veins .. .. .. .. 574

8. Question of the Life and Viability of an Infant, considered Medically and Judicially. By M. Marc .. .. .. .. .. .. .. .. .. .. .. .. .. .. .. 576

9. Cases from the Clinique of M. Bouillaud, with remarks .. .. .. .. .. .. 578

10. Sub-nitrate of Bismuth in Gastralgia.. .. .. .. .. .. .. .. .. .. .. 580

11. Practical Observations from the German Journals .. .. .. .. .. .. .. 581

12. Cases of Malignant Pustule in Man .. .. .. .. .. .. .. .. .. .. .. 582

13. On the pernicious Effects of certain Cosmetics.. .. .. .. .. .. .. .. 584

14. On the late Prevalence of Variola in London and Paris .. .. .. .. .. .. 585

15. Hysteria, Singular Case of, in which there was a Discharge of the Urine from the Umbilicus.. .. .. .. .. .. .. .. .. .. .. .. .. .. .. .. .. 586

16. On the Therapeutic Uses of Strychnine .. .. .. .. .. .. .. .. .. .. 587

17. Results of Re-Vaccination in Silesia .. .. .. .. .. .. .. .. .. .. .. 588

18. Results of Re-Vaccination in the Prussian Army, during 1836. .. .. .. .. 588

19. Treatment of Dropsy of the Bursæ Mucosæ, with Iodine .. .. .. .. .. .. 589

20. Dropsy of the Joints successfully treated with Emetics .. .. .. .. .. .. 590

21. M. Velpeau on the Treatment of Blepharitis .. .. .. .. .. .. .. .. .. 591

22. Case of Phlebitis after Venesection, with Remarks by M. Blandin.. .. .. .. 592

23. M. Chomel on Tartar Emetic in Pneumonia .. .. .. .. .. .. .. .. .. 594

24. On Paralysis in the Insane .. .. .. .. .. .. .. .. .. .. .. .. .. .. 595

25. On the Diseases of Old Age .. .. .. .. .. .. .. .. .. .. .. .. .. 597

26. M. Rostan's Remarks on Clinical Medicine .. .. .. .. .. .. .. .. .. 597

27. On the Application of Arithmetic to the Results of Therapeutics. By M. Double. 598

28. Obstinate Ague, complicated with Ascites, &c. cured with Large Doses of the Carbonate of Iron .. .. .. .. .. .. .. .. .. .. .. .. .. .. .. 601

29. Treatment of Epilepsy with the Nitrate of Silver .. .. .. .. .. .. .. .. 603

30. Observations on the Poisonous Effects of Rue, and on its Influence on the Uterus. 604

31. Researches on Menstruation .. .. .. .. .. .. .. .. .. .. .. .. .. 605

32. Case of remarkably Precocious Menstruation .. .. .. .. .. .. .. .. 606

33. Observations on Emmenagogues and on the "Aura Menstrualis.".. .. .. .. 607

34. Proto-ioduret of Mercury in Syphilis.. .. .. .. .. .. .. .. .. .. .. 607

35. On the Preparations of Silver in the Treatment of Syphilis .. .. .. .. .. 608

### Clinical Review, and Hospital Reports.

### GUY'S HOSPITAL.

1. Experiments and Observations on Albuminous Fluids. By Dr. Babington . .. 609
2. Sir A. Cooper on Varicocele of the Spermatic Cord .. .. .. .. .. .. .. 612
3. On a Morbid Flattening or Compression of the Left Bronchus, produced by Dilatation of the Left Auricle. By J. W. King .. .. .. .. .. .. .. .. 613

4. Account of a very large Calculus, passed by a young woman, without operation 614
5. On the Effect produced upon the Pulse by Change of Posture. By William Augustus Guy, M.B. .. .. .. .. .. 615
6. On Hæmorrhage from the Unimpregnated Uterus, associated with Tumors. By S. Ashwell, M.D. .. .. .. .. .. 615

BIRMINGHAM TOWN INFIRMARY.

7. Report of the Out-patients attended by F. Ryland, Esq., between 25th December, 1835, and 25th December, 1836 .. .. .. .. 619

BIRMINGHAM DISPENSARY.

8. A Report of Cases treated from January 1st, 1837, to January 1st, 1838. By T. Ogier Ward, M.D... .. .. .. .. 622

BIRMINGHAM EYE INFIRMARY.

9. A Report of Cases attended during the year 1837. By R. Middlemore, Esq. .. 624
1. Dislocation of the Lens through a rent in the Sclerotica, beneath the Conjunctiva .. .. .. .. 626
2. Employment of various Remedies for the Relief of Amaurosis .. .. 627

GENEVA HOSPITAL.

10. Report of the Cases of the In-patients treated in the Medical Wards. By W. C. Lombard, M.D. .. .. .. .. 628

PHILADELPHIA HOSPITAL.

11. Clinical Report of the Surgical Department for the Months of May, June, and July, 1837. By W. G. Horner, M.D... .. .. .. .. 629

WESTERN LYING IN HOSPITAL.

12. Second Medical Report. By Fleetwood Churchill, M.D. &c. .. .. .. .. 631

NEWRY DISPENSARY AND FEVER HOSPITAL.

13. Report for the Year 1837. By J. Morison, M.D. .. .. .. .. 632

### Miscellanies.

1. Animal Magnetism, more Miracles .. .. .. .. 634

Advent of a Mesmeric Shiloah .. .. .. .. 634

Modern Prometheus!.. .. .. .. 635

The Frankenstein Stranger .. .. .. .. 635

Transcendental Mesmerism .. .. .. .. 636

Explosion in Bedford Square .. .. .. .. 636

Death, Dissection, and Sepulture of Nickel Mania .. .. .. .. 637

2. Relationship between Epilepsy and Apoplexy. By Dr. Johnson .. .. .. .. 638

3. Bibliographial Record.. .. .. .. .. 639

### Extra-Limites.

1. On Simple Ulceration of the Stomach, with observations. By Langston Parker. M.R.C.S... .. .. .. .. 641
A. General Description of the Symptoms of Simple Ulceration of the Stomach 645
B. On the Treatment of Ulceration of the Stomach .. .. .. .. 648
2. Mr. Parker's Reclamation.. .. .. .. .. 653
3. Memorandum of the Military Medical Report from the West Indies. By W. Fergusson, M.D. .. .. .. .. 653

ST. GEORGE'S HOSPITAL.

4. On the most effectual Treatment of Acute Rheumatism in Hospital Practice during the last eight years. By Edward J. Seymour, M.D... .. .. .. .. 657
1. Acute Fibrous Rheumatism—Rheumatic Fever .. .. .. .. 657

CLARE INFIRMARY.

5. Selection of Cases from the County Clare Infirmary. By Geo. W. O'Brien, M.D. 660
6. Gleanings during three years spent in the Indian Seas. By W. B. Marshall, R.N. 666
1. New South Wales.. .. .. .. .. 666
7. Case of Rupture of the Heart. By Jas. Stephen, M.D... .. .. .. .. 671
8. Inversion and Extirpation of the Uterus .. .. .. .. 673
9. Mr. Pearson Thompson and Dr. Dickson .. .. .. .. 674

